# Potential of circulating receptor-interacting protein kinase 3 levels as a marker of acute liver injury

**DOI:** 10.1038/s41598-023-41425-6

**Published:** 2023-08-28

**Authors:** Takayuki Kondo, Kentaro Fujimoto, Kisako Fujiwara, Sae Yumita, Takamasa Ishino, Keita Ogawa, Miyuki Nakagawa, Terunao Iwanaga, Keisuke Koroki, Hiroaki Kanzaki, Masanori Inoue, Kazufumi Kobayashi, Soichiro Kiyono, Masato Nakamura, Naoya Kanogawa, Sadahisa Ogasawara, Shingo Nakamoto, Tetsuhiro Chiba, Jun Kato, Keiichi Fujiwara, Naoya Kato

**Affiliations:** 1 Department of Gastroenterology, Graduate School of Medicine, Chiba University, 1-8-1, Inohana, Chuo-ku, Chiba, 260-8670 Japan; 2 Ultrasound Center, Chiba University Hospital, Chiba, Japan

**Keywords:** Hepatology, Biomarkers

## Abstract

The pathogenesis of acute liver failure (ALF) involves cell death. Necroptosis is a newly suggested programmed cell death, and receptor-interacting protein kinase 3 (RIPK3) has been reported as a marker for necroptosis. However, there are few reports on necroptosis in ALF. Therefore, we evaluated the role of cell death markers such as cytokeratin (CK) 18, cleaved CK (cCK) 18, and RIPK3 in ALF, as well as cytokines and hepatocyte growth factor (HGF). Seventy-one hospitalized patients with acute liver injury (38 nonsevere hepatitis [non-SH]/22 severe hepatitis [SH]/11 ALF) were studied. No significant difference was found for cytokines, but a substantial increase in HGF levels was found following the severity of hepatitis. The non-SH group had lower levels of CK18 and cCK18 than the SH/ALF group. RIPK3 was significantly lower in the non-SH/SH group than in the ALF group. HGF, RIPK3, and albumin levels were found to be important predictive variables. The present study suggests that cCK18, CK18, and RIPK3 are associated with the severity of hepatitis. RIPK3 and other markers related cell death may be useful for understanding the pathogenesis of ALF and as a prognostic marker of acute liver injury.

## Introduction

Acute liver failure (ALF) is a rare condition characterized by rapid deterioration of liver function and poor prognosis in previously normal liver function^1,2^. The pathogenesis of ALF is caused by a large number of hepatocyte death in a short period, and inflammatory cells, cytokines, and damage-associated molecular patterns (DAMPs), which are inflammation-inducing molecules, spill over into the system, leading to systemic inflammation^3^. Cytokeratin (CK)18, a known marker of cell death in blood, and cleaved CK (cCK)18, a marker of apoptosis, have been reported to be associated with the severity and prognosis of chronic as well as acute hepatitis^4–10^. The emergence of organ failures in cirrhotic patients with acute decompensation (AD) defines acute-on-chronic liver failure (ACLF); a condition defined by liver cell death and a high risk of short-term mortality. With progression from AD to ACLF, the mode of cell death evolves from predominantly apoptotic to other nonapoptotic forms^11^. Necroptosis is a newly hypothesized programmed cell death that, like necrosis, causes membrane rupture, releasing DAMPs and activating surrounding inflammation^12^. There are scattered studies on the usefulness of receptor-interacting protein kinase 3 (RIPK3) as a necroptosis blood marker, despite its major role in the necroptosis pathway^13,14^. We have previously shown that plasma RIPK3 levels are associated with the progression of patients with no ACLF to ACLF, the severity of ACLF, and mortality in two separate clinical cohorts^15^. However, there are few reports on necroptosis in ALF.

Hepatocyte growth factor (HGF), a powerful hepatic mitogen, has been shown to drive liver regeneration in patients with liver failure^16,17^, and evaluation of serum HGF levels is important for prognosis prediction and disease assessment in patients with ALF and chronic liver failure^18^.

The aim of this study was to evaluate the utility of cell death markers as blood biomarkers in acute liver injury, as well as different cytokines (tumor necrosis factor-α [TNF-α], interleukin-6 [IL-6], IL-1β), and HGF.

## Patients and methods

### Patients

Serum samples and clinical data were obtained from our institutional bio-banked material and database. The study comprised 71 instances of acute liver injury who were admitted to Chiba University Hospital for treatment between December 2008 and Jun 2023 and had residual serum preservation at the time of admission. The following etiology of acute liver injury was excluded from the study: (i) infiltration of the liver by malignant cells, (ii) metabolic diseases, (iii) unclassified due to insufficient examinations, (iv) alcoholic hepatitis, and (v) circulatory disturbance. In addition, patients with chronic liver diseases except acute exacerbation of asymptomatic hepatitis B carriers were excluded, according to the Japanese criteria^2^.

The severity of hepatitis and its prognosis were examined through the blood biomarkers of CK18, cCK18, RIPK3, IL-6, IL-1β, TNF-α, and HGF using the serum preservation at the time of admission. Commercially available enzyme-linked immunosorbent assays (ELISA) were used according to the manufacturer’s instructions to measure serum levels of RIPK3 (Human RIPK3 ELISA, CUSABIO, Wuhan, China), CK18 and cCK18 (M65 ELISA and M30 Apoptosense respectively, Peviva, Tewkesbury, UK), TNF-α, IL-6, IL-1β, and HGF (R&D systems, Minneapolis, USA). Absorbance was determined using a multi-mode plate reader (FLUOstar Omega, BMG Labtech, UK). The intraclass correlation coefficient with the ELISA results of the remaining serum stores was as high as 0.732 in the 54 patients who had HGF testing done upon admission. The data were adjusted using the correction equation obtained in the linear regression model.

### Definitions

ALF was defined as patients with hepatic encephalopathy (HE) within eight weeks of the onset of illness and coagulation abnormality (prothrombin time [PT] values of 40% or less of the standardized values or international normalized ratios of 1.5 or more), according to the Japanese diagnostic criteria of ALF^2^. Patients with coagulation abnormality but without HE were classified as having severe hepatitis (SH). ALF is further classified into two disease types, the acute type and subacute type, with HE developing within ten days and between 11 and 56 days after the onset of disease symptoms, respectively^19^. HE was assessed using the West Haven classification system^20^, and this study defined grade II or higher as HE in ALF, using the Japanese criteria^2^. Viral infection, autoimmune hepatitis (AIH), drug-induced liver injury (DILI), and indeterminate causes were the categories used to categorize the causes of hepatitis^19^. A diagnosis of viral infection was made based on the positivity of for viral antibody. AIH was diagnosed based on the presence of antinuclear antibody and/or anti-smooth muscle antibody, as well as the international Autoimmune Hepatitis Group^21^ definite or probable diagnosis. DILI was diagnosed based on its clinical features and courses. All patients received comprehensive supportive care, and some received corticosteroid (CS) pulse therapy, consisting of 1000 mg or 500 mg methylprednisolone (MPSL) daily for three days, followed by a lower dose of MPSL based on treatment response^22^.

### Statistical analysis

All data are expressed as means ± standard deviation or as percentages. The Student's t-test, the Mann–Whitney U-test, Fisher’s protected least significant difference test, or Kruskal–Wallis test was used to analyze continuous variables, while the chi-squared test was used to analyze categorical variables, as appropriate. The multivariate analysis was assessed by logistic regression analysis. The correlation coefficient (*r*) was calculated using either Spearman’s rank correlation coefficient or Pearson correlation coefficient, depending on the situation. Classification and regression tree (CART) analysis was performed using the R-powered data tool Exploratory (https::/exploratory.io)^23^. Furthermore, P < 0.05 was also considered significant, and statistical data were analyzed using SAS version 9.2 (SAS Institute, Cary, NC).

### Animal research (ethics)

This study does not contain animal experimental data.

### Ethics and consent to participate

This study conformed to the principles of the Declaration of Helsinki and was approved by the Ethics Committee of Chiba University Graduate School of Medicine. The Ethics Committee of Chiba University Graduate School of Medicine approved that written informed consent was waived because of the retrospective design, and informed consent was obtained in the form of an opt-out on the web-site.

## Results

### Patient characteristics

The characteristics of the subject are summarized in Table 1. Among the 71 patients, the etiology was due to viral infection in 22, DILI in 19, AIH in 17, and indeterminate causes in 13. Thirty-eight cases were non-SH, 22 cases of SH, and 11 cases of ALF (4 acute and 7 subacute). All patients with ALF received an infusion of fresh frozen plasma and high-flow dialysate continuous hemodiafiltration (HF-CHDF). Ten patients (90.9%) with ALF and 14 patients (63.6%) with SH received CS pulse therapy.

**Table 1:** Patient characteristics.

	Non-severe hepatitis (N = 38)	Severe hepatitis (N = 22)	Acute liver failure (N = 11)
Age (years)	50.1 ± 18.5	45.5 ± 16.6	54.7 ± 14.6
Male	27 (71.1%)	9 (40.9%)	5 (45.5%)
Body mass index	22.9 ± 3.6	22.6 ± 3.3	25.4 ± 3.8
Etiology (AIH/drug/virus/indeterminate)	11/10/13/4	3/7/6/6	3/2/3/3
Ascites	6 (15.8%)	7 (31.8%)	7 (63.6%)
Laboratory data			
Aspartate aminotransferase (U/L)	751 ± 680	2548 ± 3505	1564 ± 1852
Alanine aminotransferase (U/L)	1119 ± 971	2479 ± 3120	2172 ± 2902
Bilirubin (mg/dL)	8.3 ± 7.3	13.7 ± 9.5	19.2 ± 18.2
Prothrombin time (%)	76.8 ± 22.6	30.2 ± 9.8	19.1 ± 8.1
Fresh frozen plasma	0	10 (45.5%)	11 (100%)
High-flow dialysate continuous hemodiafiltration	0	0	11 (100%)
Corticosteroid pulse therapy	7 (18.4%)	14 (63.6%)	10 (90.9%)

Data are expressed as mean ± standard deviation or number (%).

*AIH* autoimmune hepatitis.

### Hepatitis severity and various biomarkers

**Figure 1:**
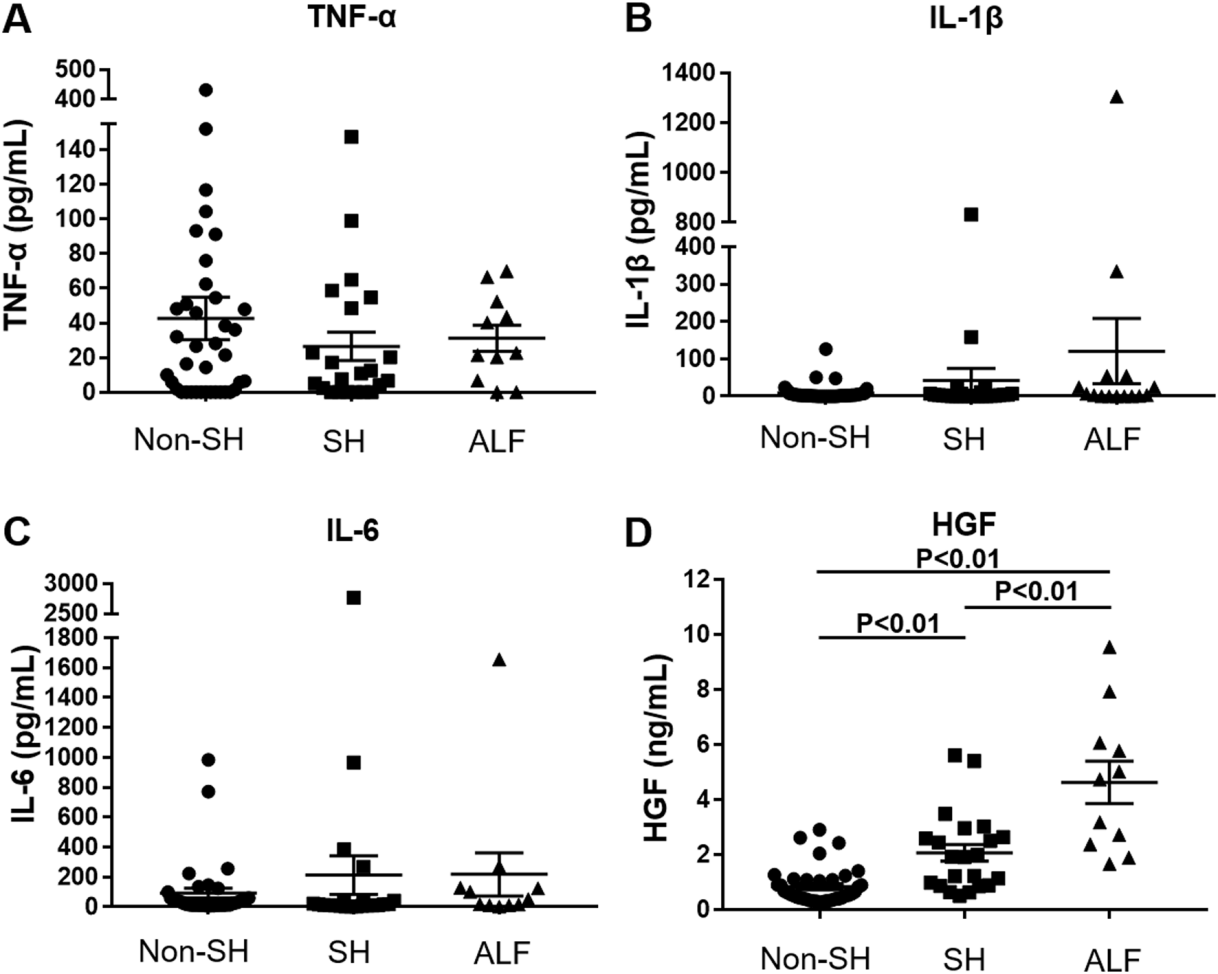
Serum levels of TNF-α, IL-1β, IL-6, and HGF stratified by hepatitis severity (38 cases of non SH, 22 cases of SH, 11 cases of ALF). (**A**) There were no significant differences in serum levels of TNF-α by hepatitis severity. (**B**) There were no significant differences in serum levels of IL-1β by hepatitis severity. (**C**) There were no significant differences in serum levels of IL-6 by hepatitis severity. (**D**) As for serum levels of HGF, there was a significant increase consistent with the severity of hepatitis (P < 0.01). *ALF* acute liver failure, *HGF* hepatic growth factor, *IL-1β* interleukin-1β, *IL-6* interleukin-6, *non-SH* nonsevere hepatitis, *SH* severe hepatitis, *TNF-α* tumor necrosis factor-α.

There were no significant differences in serum levels of TNF-α, IL-6, and IL-1β by hepatitis severity (Fig. 1). Furthermore, although only a small number of patients, we examined changes in serum levels of TNF-α, IL-6, and IL-1β after CS pulse therapy in 13 patients and discovered that serum TNF-α levels improved promptly after CS pulses in 7 of the 13 patients and that serum IL-6 levels decreased promptly after CS pulse therapy in 10 of the 13 patients. However, no specific changes in serum IL-1β levels were observed after CS pulse therapy (Supplementary Fig. S1). As for serum HGF levels, there was a significant increase consistent with the severity of hepatitis (non-SH: 0.8 ± 0.7 ng/mL, SH: 2.1 ± 1.4 ng/mL, ALF: 4.6 ± 2.6 ng/mL, P < 0.01) (Fig. 1).

**Figure 2:**
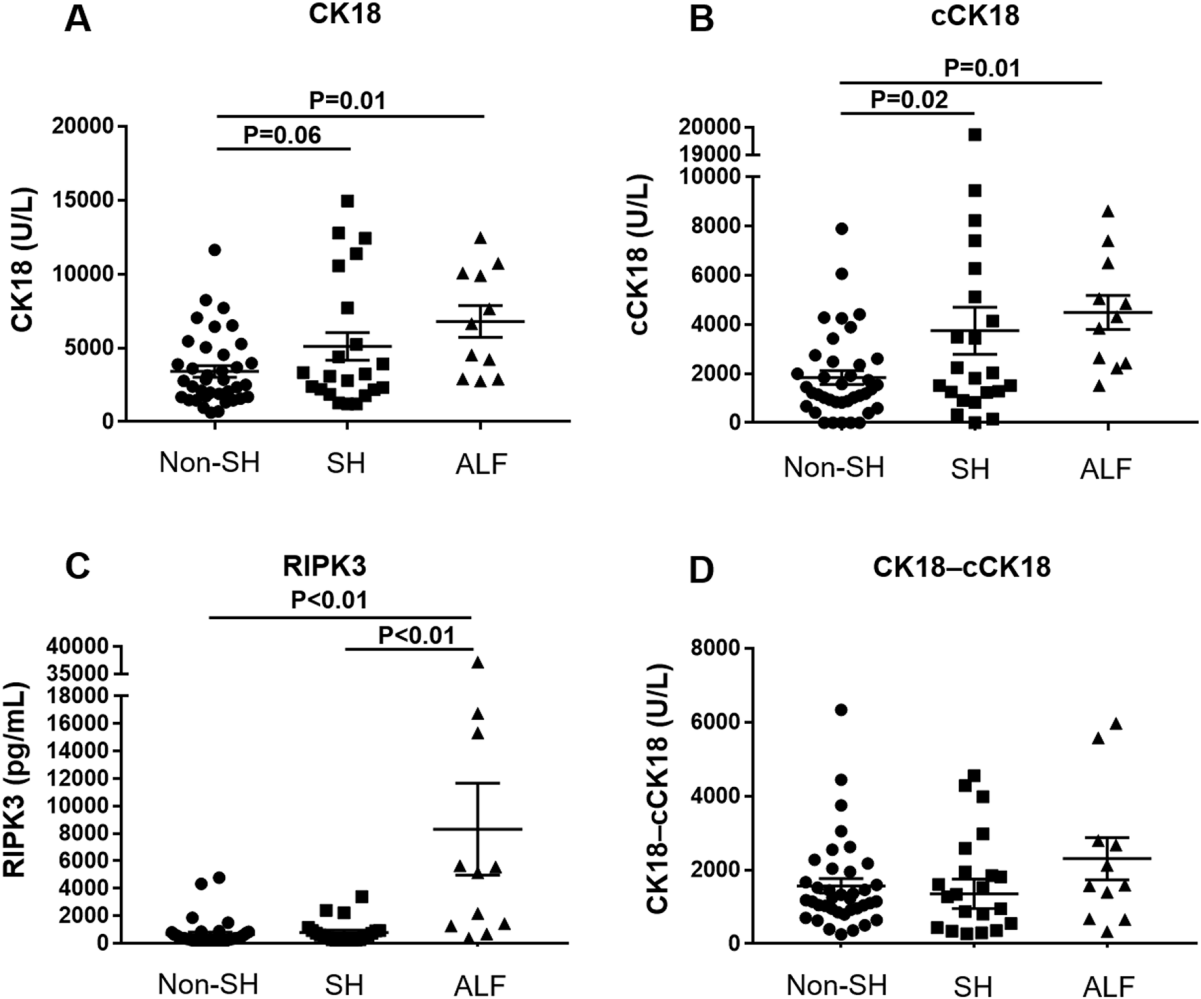
Serum levels of CK18, cCK18, RIPK3, and CK18-cCK18 were stratified by hepatitis severity (non-SH [N = 38] *vs*. SH [N = 22] *vs*. ALF [N = 11]). (**A**) Serum CK18 levels were significantly higher in the ALF group than in the non-SH group (P = 0.01), and showed a trend toward higher in the SH group than in the non-SH group (P = 0.06). (**B**) Serum cCK18 levels were significantly higher in the SH (P = 0.02)/ALF group (P = 0.01) than in the non-SH group. (**C**) Serum RIPK3 levels were significantly lower in the non-SH/SH group compared to the ALF group (P < 0.01). (**D**) CK18-cCK18 levels showed no significant difference between 3 groups, but a trend toward higher in the ALF group than in the non-SH (P = 0.17)/SH groups (P = 0.10). *ALF* acute liver failure, *cCK18* cleaved cytokeratin 18, *CK18* cytokeratin 18, *non-SH* nonsevere hepatitis, *SH* severe hepatitis, *RIPK3* receptor-interacting protein kinase 3.

**Figure 3:**
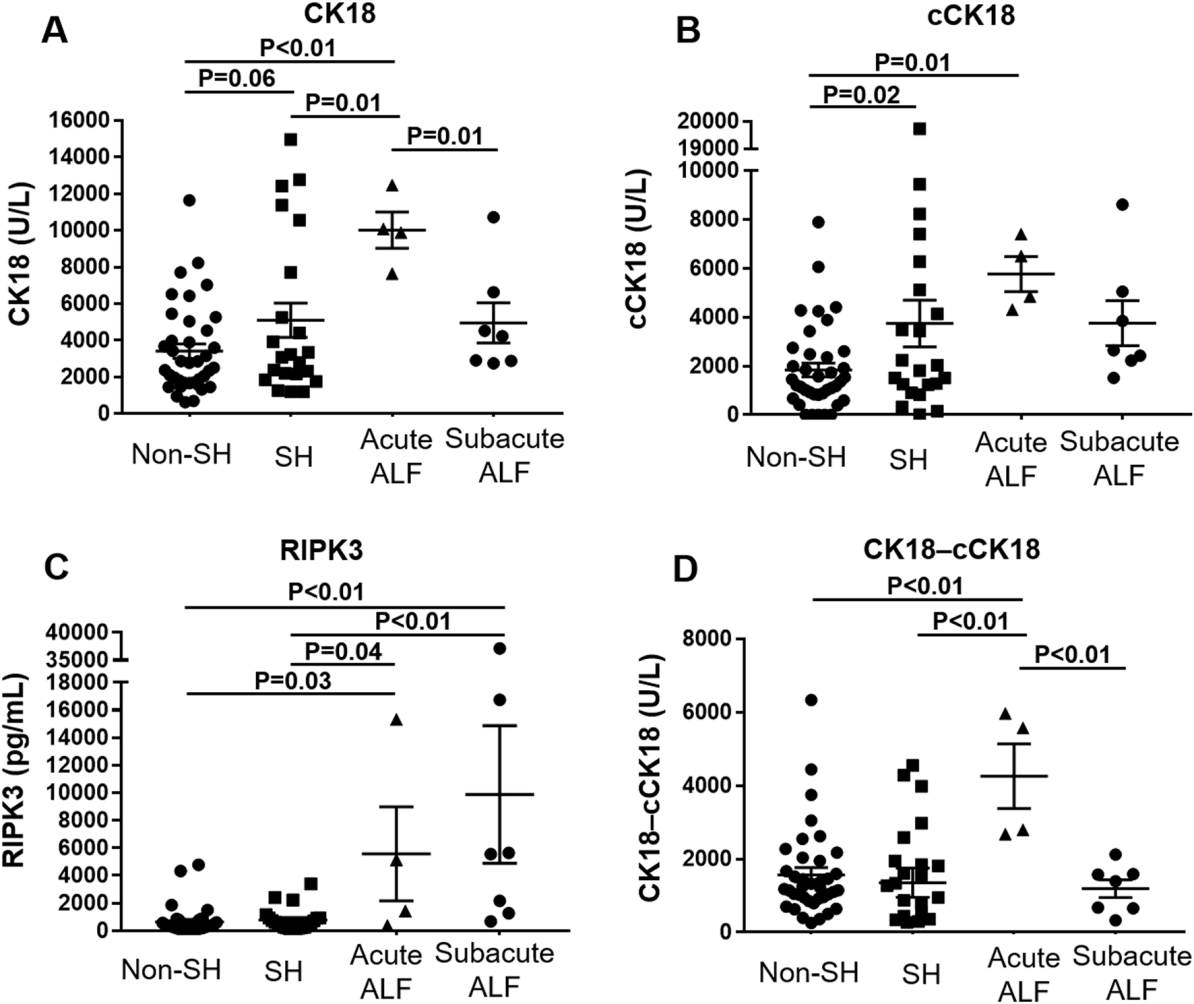
Serum levels of CK18, cCK18, RIPK3, and CK18-cCK18 were stratified by hepatitis severity (non-SH [N = 38] *vs*. SH [N = 22] vs. acute ALF [N = 4] *vs*. subacute ALF [N = 7]). When subdivided ALF into acute and subacute ALF. (**A**) CK18 levels were significantly lower in the subacute ALF group compared to the acute ALF group (P = 0.01). (**B**) There were no significant differences in cCK 18 levels between acute and subacute ALF groups. (**C**) RIPK3 levels were higher in the acute/subacute ALF groups than in the non-SH/SH groups (non-SH *vs*. acute ALF: P = 0.03; non-SH *vs*. subacute ALF: P < 0.01; SH *vs*. acute ALF: P = 0.04; SH *vs*. subacute ALF; P < 0.01), and showed a trend toward higher in the subacute ALF group than in the acute ALF group (P = 0.11). (D) CK18-cCK18 levels showed higher levels in the acute ALF group than in the non-SH/SH/subacute ALF groups (P < 0.01). *ALF* acute liver failure, *cCK18* cleaved cytokeratin 18, *CK18* cytokeratin 18, *non-SH* non-severe hepatitis, *SH* severe hepatitis, *RIPK3* receptor-interacting protein kinase 3.

Serum CK18 levels, a marker of cell death including apoptosis and necrosis, and serum cCK18 levels, a marker of apoptosis, were higher in the SH/ALF group than in the non-SH group (Fig. 2). A marker of necrosis, subtracting cCK18 levels from CK18 (CK18-cCK18) levels^24^, showed no significant difference between 3 groups, but a trend toward higher in the ALF group than in the non-SH/SH groups (non-SH *vs.* ALF:1572 ± 1203 *vs.* 2306 ± 1889, P = 0.17; SH *vs.* ALF: 1357 ± 1887 vs. 2306 ± 1889, P = 0.10; Fig. 2). Serum RIPK3 levels, a possible marker of necroptosis, were significantly lower in the non-SH/SH group compared to the ALF group (non-SH *vs.* ALF: 649 ± 1012 pg/mL *vs.* 8317 ± 11,100 pg/mL, P < 0.01; SH *vs.* ALF: 784 ± 856 *vs.* 8317 ± 11,100 pg/mL, P < 0.01) (Fig. 2). When subdivided ALF into acute and subacute ALF, CK18 levels were significantly lower in the subacute ALF group compared to the acute ALF group, in contrast to RIPK3 levels (Fig. 3). CK18-cCK18 levels showed higher levels in the acute ALF group than in other 3 groups (Fig. 3).

### Prognosis factors

Nine patients (12.7%) died while they were hospitalized. The levels of several biomarkers and clinical results were compared between the survival and death group.

In univariate analysis, serum levels of cytokines, CK18 and cCK18 were not related to prognosis, but serum HGF levels (survival *vs.* death: 1.4 ± 1.3 ng/mL *vs.* 4.6 ± 2.8 ng/mL, P < 0.01) and serum RIPK3 levels (survival *vs.* death: 837 ± 1259 pg/mL *vs.* 9055 ± 12,236 pg/mL, P < 0.01) were significantly higher in the death group than the survival group (Table 2). Regarding the clinical findings, univariate analysis revealed that the significant predictive markers included indeterminate etiology, high model for end-stage liver disease (MELD) score, high total bilirubin levels, low albumin levels, high ammonia levels, high white blood cell levels, low PT, and the presence of ascites (Table 2). However, no significant factors were observed in the multivariate analysis.

**Table 2:** Comparisons of serum biomarkers and clinical findings between survival and death group during hospitalization.

	Survival (N = 62)	Death (N = 9)	*P* value
Serum biomarkers			
TNF-α (pg/mL)	36.4 ± 63.6	32.2 ± 24.6	0.85
IL-6 (pg/mL)	132.5 ± 395.1	266.3 ± 527.6	0.37
IL-1β (pg/mL)	28.6 ± 114.7	156.5 ± 431.8	0.05
HGF (ng/mL)	1.4 ± 1.3	4.6 ± 2.8	< 0.01
CK18 (U/L)	4495 ± 3607	4247 ± 2804	0.84
cCK18 (U/L)	2877 ± 3279	2619 ± 1454	0.82
RIPK3 (pg/mL)	837 ± 1259	9055 ± 12,236	< 0.01
Clinical findings			
Age (years)	48.0 ± 17.0	58.9 ± 18.5	0.08
Male	36 (58.1%)	5 (55.6%)	0.89
Body mass index	22.9 ± 3.5	25.3 ± 3.4	0.10
Etiology (autoimmune hepatitis)	15 (24.2%)	2 (22.2%)	0.90
Etiology (drug)	17 (27.4%)	2 (22.2%)	0.17
Etiology (virus)	21 (33.9%)	1 (11.1%)	0.17
Etiology (Indeterminate)	9 (14.5%)	4 (44.4%)	0.03
Ascites	13 (21.0%)	7 (77.8%)	< 0.01
Laboratory data			
Aspartate aminotransferase (U/L)	1563 ± 2380	542 ± 329	0.21
Alanine aminotransferase (U/L)	1874 ± 2349	526 ± 405	0.09
Bilirubin (mg/dL)	9.9 ± 8.4	23.7 ± 9.9	< 0.01
Prothrombin time (%)	57.6 ± 30.2	21.3 ± 9.9	< 0.01
Lactate dehydrogenase (U/L)	788 ± 1285	599 ± 576	0.67
Albumin (g/dL)	3.5 ± 0.6	2.5 ± 0.4	< 0.01
Ammonia (μg/dL)	62.6 ± 34.8	11.7 ± 71	< 0.01
Creatinine (mg/dL)	0.87 ± 1.16	1.36 ± 0.74	0.23
Sodium (mmol/L)	137 ± 4	134 ± 5	0.09
C-reactive protein (mg/dL)	1.16 ± 1.58	1.42 ± 1.21	0.63
White blood cell (× 10^9^/L)	6.5 ± 2.8	12.2 ± 5.8	< 0.01
Platelets (10^9^/L)	189 ± 88	145 ± 56	0.15
MELD	18.3 ± 6.6	30.7 ± 5.5	< 0.01

Data are expressed as mean ± standard deviation or number (%).

The Student’s t-test, or the Mann–Whitney U-test was used to analyze continuous variables, while the chi-squared test was used to analyze categorical variables.

*cCK18* cleaved cytokeratin 18, *CK18* cytokeratin 18, *HGF* hepatic growth factor, *IL-1β* interleukin-1β, *IL-6* interleukin-6, *RIPK3* receptor-interacting protein kinase 3, *MELD* model for end-stage liver disease, *TNF-α* tumor necrosis factor-α.

To construct a prognostic model, the most relevant variables were chosen for CART analysis, and several trees were constructed using an exploratory strategy. The final tree-discriminated cases were divided into four groups with distinct prognoses: very poor prognosis (albumin < 2.7 g/dL and HGF ≥ 2.7 ng/mL, mortality rate: 100%), poor prognosis (albumin ≥ 2.7 g/dL and RIPK3 ≥ 5357 pg/dL, mortality rate 67%), fair prognosis (albumin < 2.7 g/dL and HGF < 2.7 ng/mL, mortality rate 20%), and good prognosis (albumin ≥ 2.7 g/dL and RIPK3 < 5357 pg/mL, mortality rate: 0%).

### Correlation of serum biomarkers and clinical findings with serum RIPK3

Table 3 shows the relationship between other biomarkers and clinical findings on serum RIPK3 levels. Serum RIPK3 concentrations showed a positive correlation with serum HGF concentrations (*r* = 0.65, P < 0.01), serum CK18 concentrations (*r* = 0.34, P < 0.01), ascites presence (*r* = 0.24, P = 0.04), total bilirubin concentrations (*r* = 0.31, P = 0.01), ammonia concentrations (*r* = 0.55, P < 0.01), creatinine concentrations (*r* = 0.38, P < 0.01), and C-reactive protein (CRP) (*r* = 0.38, P < 0.01). Albumin concentrations (*r* = -0.31, P = 0.01) showed a negative correlation with RIPK3. However, serum RIPK3 levels did not show any correlation with serum cCK18 levels, a maker of apoptosis, and serum levels of cytokines. MELD score showed a positive correlation with serum RIPK3 (r = 0.57, P < 0.01), CK18 (r = 0.27, P = 0.02), cCK18 (r = 0.30, P = 0.01), and HGF (r = 0.75, P < 0.01).

**Table 3:** Correlation of serum biomarkers and clinical findings with serum RIPK3.

	Serum RIPK3 (pg/mL)	
	Correlation coefficient (*r*)	*P* value
Serum biomarkers		
TNF-α (pg/mL)	− 0.03	0.82
IL-6 (pg/mL)	− 0.01	0.98
IL-1β (pg/mL)	− 0.02	0.84
HGF (ng/mL)	0.65	< 0.01
CK18 (U/L)	0.34	< 0.01
cCK18 (U/L)	0.05	0.65
Clinical findings		
Age (years)	0.02	0.76
Male	− 0.02	0.84
Body mass index	0.08	0.54
Etiology (autoimmune hepatitis)	− 0.12	0.30
Etiology (drug)	0.10	0.41
Etiology (virus)	− 0.04	0.77
Etiology (Indeterminate)	0.07	0.58
Ascites	0.24	0.04
Laboratory data		
Aspartate aminotransferase (U/L)	− 0.05	0.69
Alanine aminotransferase (U/L)	− 0.04	0.73
Bilirubin (mg/dL)	0.31	0.01
Prothrombin time (%)	− 0.24	0.04
Lactate dehydrogenase (U/L)	− 0.04	0.73
Albumin (g/dL)	− 0.31	0.01
Ammonia (μg/dL)	0.55	< 0.01
Creatinine (mg/dL)	0.32	0.01
Sodium (mmol/L)	− 0.08	0.54
C-reactive protein (mg/dL)	0.38	< 0.01
White blood cell (× 10^9^/L)	0.19	0.12
Platelets (10^9^/L)	− 0.06	0.64
MELD	0.57	< 0.01

The correlation coefficient (r) was calculated using either Spearman’s rank correlation coefficient or Pearson correlation coefficient.

*cCK18* cleaved cytokeratin 18, *CK18* cytokeratin 18, *HGF* hepatic growth factor, *IL-1β* interleukin-1β, *IL-6* interleukin-6, *MELD* model for end-stage liver disease, *RIPK3* receptor-interacting protein kinase 3, *TNF-α* tumor necrosis factor-α.

## Discussion

The present study suggests that CK18, cCK18, and RIPK3, markers of cell death, are associated with hepatitis severity and MELD in a small number of cases. CK18, a marker that includes apoptosis, necrosis, and other cell death, was associated with RIPK3, a marker of necroptosis, but no association was seen with cCK18, a marker of apoptosis. This will suggest that RIPK3 is an independent marker of cell death distinct from apoptosis. In particular, the fact that CK18-cCK18 levels were raised in acute ALF suggests that the proportion of nonapoptotic cell death has increased in the acute ALF. The fact that RIPK3 was significantly higher in the acute ALF than non-SH and SH lend credence to this theory. In other words, the percentage of apoptosis, a type of nonapoptotic cell death, may be comparatively higher in the acute ALF than the percentage of apoptosis. Interestingly, cell death markers were markedly reduced in subacute ALF, which is expected because most of hepatocytes are already dead in subacute ALF. However, circulating RIPK3 was elevated in subacute ALF in contrast to CK18 and cCK18, suggesting that RIPK3 may not be the only simple marker of necroptosis. It is reported that acute severe liver injury impairs hepatocyte regeneration through the induction of senescence^25^, and RIPK3 and caspase-8 induced T-cell senescence^26^. Further studies are needed to determine whether RIPK3 is also a marker of liver regeneration and senescence. This has not been reported previously and is considered to be an important marker for understanding the pathogenesis of ALF.

The most significant finding in this study was the possibility that circulating RIPK3 could serve as a biomarker for acute liver injury. However, the mechanism and source of the circulating RIPK3 are unclear. The liver is thought to be the primary source of elevated circulating RIPK3 in ACLF, but the kidneys may also be a source, as we have previously demonstrated^15^. In the current study, RIPK3 not only correlated with markers of the hepatic functional reserve but also with renal function. This is in line with circulating RIPK3, which has been identified as a marker of acute kidney injury in critically ill patients after trauma^13^. In addition, there was an association between elevated circulating RIPK3 and CRP, which are indicators of inflammation and infection. However, there was no evidence linking circulating RIPK3 to cytokines. One possible explanation for these findings is that the study included patients with an acute liver injury who required hospitalization, and the majority of their immune systems were activated at the time of admission. In an ACLF report, it is stated that CRP levels at the time of admission are not a suitable prognostic indicator due to high CRP levels in patients without ACLF^27,28^, even though the main pathogenesis of ACLF is inflammation^29^, but it is the persistence of high CRP levels after admission that is associated with prognosis^28^. Therefore, it may also be important to prevent the persistence of high cytokine levels in acute liver injury. The results of randomized controlled trials in Europe and the United States in the 1970s^30,31^ ruled out the use of CS for ALF, but as previously reported , immunosuppressive therapy with a focus on CS has been associated with suppression of hepatocyte death and improvement outcome of ALF^32,33^. In Japan, CS is administered in more than 70% of cases, as they are thought to inhibit death^34^. We previously reported on the possibility of a decrease in cytokines following CS administration^22^, and in the current study, we observed a trend in cytokines following CS administration in a small number of cases, indicating the possibility of an early decrease in inflammation following CS administration. However, necroptosis is induced by a variety of stimuli, including TNFα, Fas ligand, lipopolysaccharide, TNF-related apoptosis-inducing ligand, and interferon α/β, and accelerates inflammation^35,36^. In addition, RIPK3 induces inflammation independent of necroptosis^37^. Elevated plasma RIPK3 levels may be a sign of ongoing inflammation following hospitalization. A study should be performed to clarify the indications for CS administration as well as the indicators to determine CS therapy continuation, taking into account the state of liver injury and cell death.

Apoptosis is considered to suppress cytokines and subsequent immune response^36^. As a result, CK18 and cCK18, which reflect apoptosis, may not reflect prognosis in acute liver injury, where cell death is more common. Serum HGF is elevated in fulminant hepatitis, and its measurement is known to be useful in predicting fulminant hepatitis and its prognosis^38,39^. HGF was found to be well correlated with the severity of hepatitis and its prognosis in this study, with the group with high HGF and high RIPK3 having a high probability of death. This suggests that blood tests performed at the time of admission may be able to predict the need for a future liver transplant. Furthermore, low albumin levels were associated with poor prognosis. Hypoalbuminemia is known to be important as an indicator of inflammation and hepatic reserve^40,41^. From a different perspective, it is difficult to completely distinguish ALF from ACLF, because information on pre-existing liver damage is often lacking^42,43^, and this study may not have completely ruled out pre-existing chronic hepatitis or liver failure. In addition, RIPK3 has also been reported to be significantly elevated in ACLF^15^. Circulating RIPK3 and albumin levels may have the potential to reflect chronic hepatitis or liver failure prior to hospitalization, and further studies are needed to differentiate ALF from ACLF.

There are some limitations to our paper. First, as the number of data is small, this study is preliminary and has the possibility of misinterpretation. Second, no information is available on pyroptosis, which is an inflammatory cell death caused by intracellular sensors like Nod-like receptor protein 3^36^. Therefore, large-scale studies that include pyroptosis are thus desirable for understanding the pathogenesis of ALF and developing new therapeutic strategies. In addition, this study used the Japanese diagnostic criteria of ALF. The major difference between the most widely accepted definition of ALF and the Japanese criteria is the definition of HE in ALF. The Japanese criteria adopt grade II or more of HE, while the global criteria adopt any HE grades^1,43^. Therefore, we re-diagnosed all patients using the global criteria of ALF. However, in our study, patients with a minimal change in the level of consciousness promptly progressed to grade II or more of HE, so no new additional cases were diagnosed with ALF using the global criteria.

In conclusion, the balance of cell death may change as a condition progresses to ALF, while the proportion of nonapoptotic cell death, such as necroptosis, may increase. Furthermore, not only HGF but also other markers related to cell death may be useful as blood biomarkers in acute liver injury.

### Supplementary Information

Supplementary Figure 1.

## Data Availability

All data generated or analysed during this study are included in this published article.

## Abbreviations

ACLF: Acute-on-chronic liver failure
AD: Acute decompensation
AIH: Autoimmune hepatitis
ALF: Acute liver failure
CART: Classification and regression tree
cCK: Cleaved cytokeratin
CK: Cytokeratin
CS: Corticosteroid
DAMPs: Damage-associated molecular patterns
DILI: Drug-induced liver injury
ELISA: Enzyme-linked immunosorbent assays
HE: Hepatic encephalopathy
HF-CHDF: High-flow dialysate continuous hemodiafiltration
HGF: Hepatocyte growth factor
IL: Interleukin
INR: International normalized ratio
Non-SH: Nonsevere hepatitis
MELD: Model for end-stage liver disease
MSPL: Methylprednisolone
PT: Prothrombin
SH: Severe hepatitis
TNF: Tumor necrosis factor
RIPK3: Receptor-interacting protein kinase
