# Supplementary material for: Potential of circulating receptor-interacting protein kinase 3 levels as a marker of acute liver injury
Source: Sci Rep. 2023 Aug 28;13:14043. doi: 10.1038/s41598-023-41425-6 (PMC10462689; doi:10.1038/s41598-023-41425-6)
Supplement: Supplementary file 1 — Supplementary Figure 1. [file 41598_2023_41425_MOESM1_ESM.pdf]

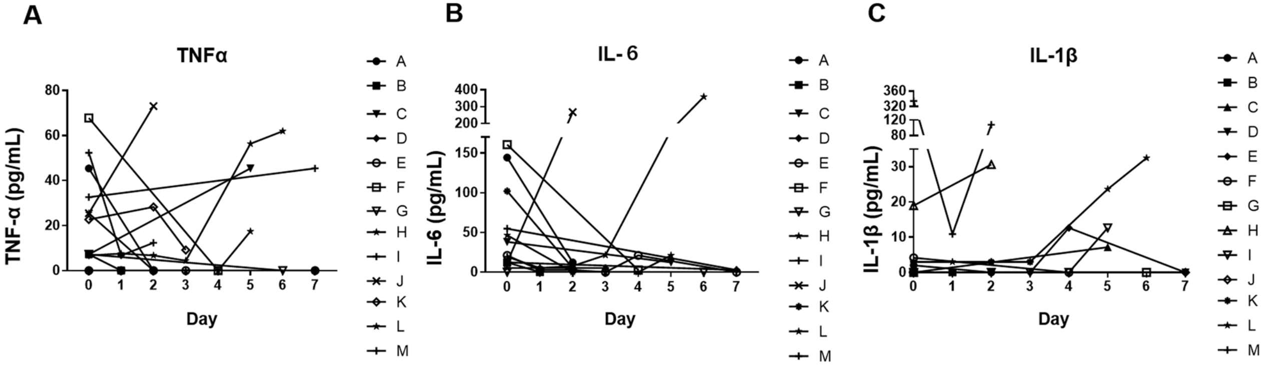

Supplementary Figure 1. Changes in serum levels of TNF-α, IL-6, IL-1β after corticosteroid pulse therapy (N = 13).
(A) Serum TNF-α levels improved promptly after CS pulses in 7 of the 13 patients. (B) Serum IL-6 levels decreased promptly after CS pulse therapy in 10 of the 13 patients. (C) No specific changes in serum IL-1β levels were observed after CS pulse therapy.
IL-1β: interleukin-1β; IL-6: interleukin-6; TNF-α: tumor necrosis factor-α.
